# Postnatal depletion of maternal cells biases T lymphocytes and natural killer cells’ profiles toward early activation in the spleen

**DOI:** 10.1242/bio.059334

**Published:** 2022-11-09

**Authors:** Flore S. Castellan, Naoki Irie

**Affiliations:** Department of Biological Sciences, Graduate School of Science, University of Tokyo, Hongo, Bunkyo-ku, Tokyo 113-0033, Japan

**Keywords:** Microchimerism, Maternal Cells, Neonatal Immunity

## Abstract

The maternal cells transferred into the fetus during gestation persist long after birth in the progeny. These maternal cells have been hypothesized to promote the maturation of the fetal immune system *in utero* but there are still significant gaps in our knowledge of their potential roles after birth. To provide insights into these maternal cells’ postnatal functional roles, we set up a transgenic mouse model to specifically eliminate maternal cells in the neonates by diphtheria toxin injection and confirmed significant depletion in the spleens. We then performed immunophenotyping of the spleens of two-week-old pups by mass cytometry to pinpoint the immune profile differences driven by the depletion of maternal cells in early postnatal life. We observed a heightened expression of markers related to activation and maturation in some natural killer and T cell populations. We hypothesize these results to indicate a potential postnatal regulation of lymphocytic responses by maternal cells. Together, our findings highlight an immunological influence of maternal microchimeric cells postnatally, possibly protecting against adverse hypersensitivity reactions of the neonate at a crucial time of new encounters with self and environmental antigens.

## Teaser

**Summary:** After specific depletion of maternal cells in neonates using diphtheria toxin receptor transgenic mice, early activation of splenic T and natural killer cells was observed.

## INTRODUCTION

In placental mammals, the transfer of cells from the mother to the fetus during pregnancy results in the presence of a small number of maternal cells persisting in the host long after birth – a phenomenon known as maternal microchimerism (MMc) (Maloney et al., 1999; Vernochet et al., 2007). The detection of immune MMc cells in the lymphoid organs in fetal and neonatal stages has raised the question of a potential impact on the development of the host's immune system (Piotrowski and Croy, 1996; Stelzer et al., 2015). In humans, MMc has been suggested to promote fetal immune tolerance and to prevent materno-fetal conflicts, as human fetal immune cells are known to be capable of alloreactivity (Mold et al., 2008). However, the murine immune system is relatively immature until birth (Mold and McCune, 2012) and inducing fetal tolerance toward MMc may be less necessary for pregnancy maintenance. Further immune functional roles of MMc may help explain the presence and retention after birth of maternal cells in mice.

Contrasting consequences of MMc on the progeny immune system have been proposed. As a beneficial effect, MMc was speculated to be able to stimulate the maturation of the nascent immune system (Kinder et al., 2017), with promising results suggesting MMc promotes monocytes and T cells’ differentiation and responses (Balle et al., 2022; Stelzer et al., 2021). However, while mutual tolerance is the norm, scarce MMc-induced immune perturbations may lead to auto- or alloreactive immune conflicts (Leveque et al., 2014; Müller et al., 2001) – an etiopathogenesis formulated as the materno-fetal immune disease hypothesis (Irie et al., 2009; Muraji et al., 2009).

Previous research designed to assess the immune functions of maternal cells conducted experiments with a reduced number of MMc cells, by using mice deficient in maternal lymphocytes from conception (Stelzer et al., 2021) or antibody-mediated MMc elimination in adulthood (Kinder et al., 2015). To extend the current knowledge on MMc functions to their postnatal immune roles, we implemented an approach to deplete maternal cells in the neonates by diphtheria toxin (DT) injection and compared the spleens’ immune profiles of the 2-week-old pups with intact and depleted maternal cells by mass cytometry immunophenotyping. We believe depleting maternal cells shortly after birth may shed light on some potential functional roles in a particularly immune-demanding developmental period.

## RESULTS

### Mouse breeding schema for maternal cells' depletion and detection

**Fig. 1.**
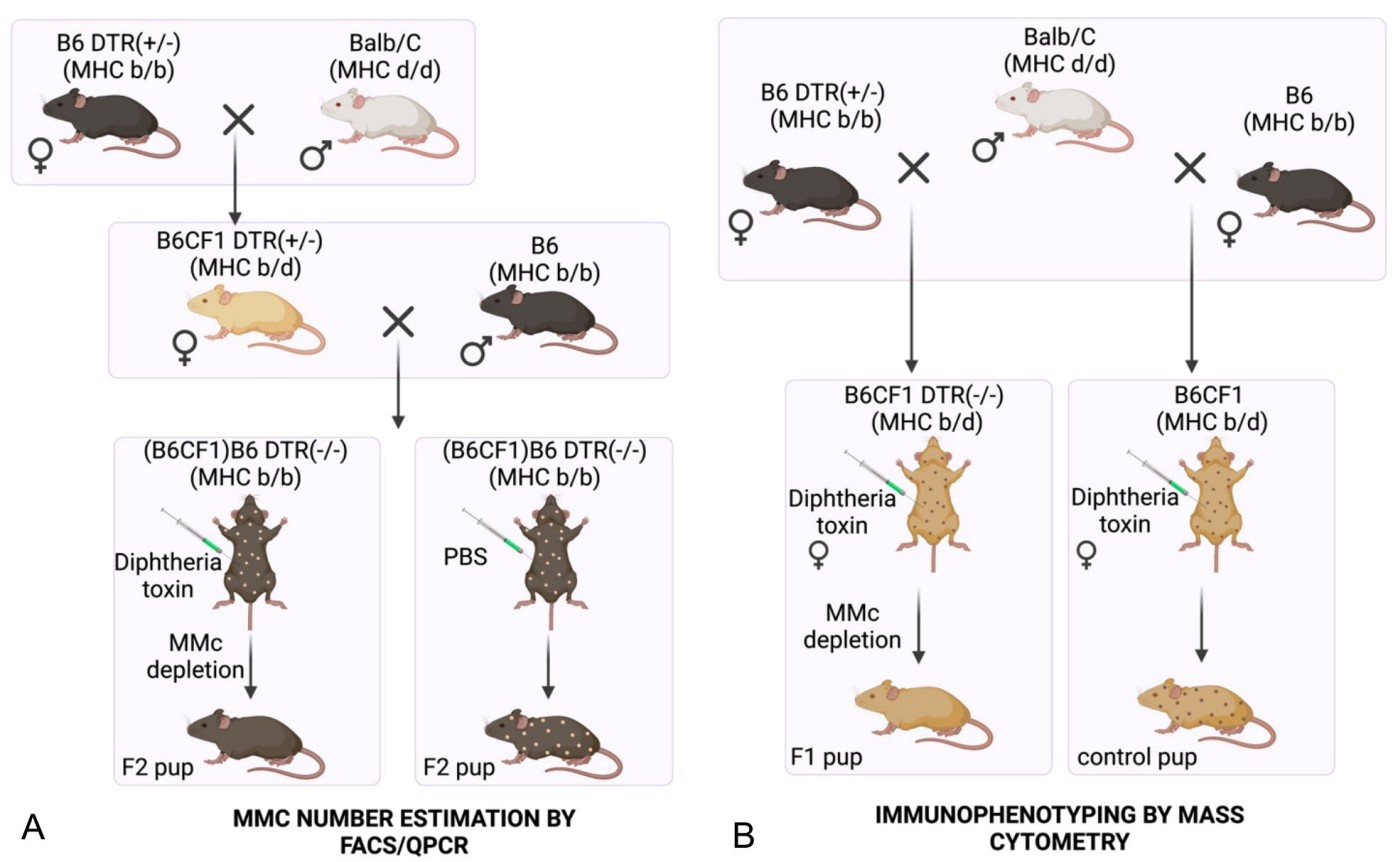
**Mouse breeding charts.** (A) Mouse crossings to obtain the F2 pups in which DTR(+/-)MHC(b/d) maternal cells numbers were estimated by FACS/qPCR. (B) Mouse crossings to obtain the F1 and control pups whose spleens were immunophenotyped by mass cytometry. B6 and Balb/C refer to the C57BL/6JJcl and BALB/cByJJcl background strains, and B6CF1 and (B6CF1)B6 refer to hybrid backgrounds from crossing these strains. The major histocompatibility complex (MHC) alleles of each mouse are indicated in parenthesis. DTR: diphtheria toxin receptor. MMc: maternal microchimerism. Created with BioRender.com.

To deplete maternal cells in the pups, we took advantage of the targeted apoptotic depletion system in diphtheria toxin receptor (DTR) transgenic mice. By crossing the iDTR strain (Buch et al., 2005) with CAG-Cre mice, we first obtained mice with ubiquitous expression of DTR. In wild-type DTR(−/−) pups born from heterozygous DTR(+/−) dams, transgenic DTR(+/−) maternal cells could be specifically targeted by diphtheria toxin (DT)-induced apoptosis via diphtheria toxin injections. For the detection of maternal cells in the pups, DTR(+/−) dams on the B6CF1 hybrid genetic background with heterozygous major histocompatibility complex (MHC) b/d were used as the source of maternal cells in backcrossed DTR(−/−) MHC b/b pups (F2 pups, Fig. 1A). With this configuration, maternal cells could be distinguished among the pups’ cells by both DTR and MHCd expressions, and their depletion could be tested.

For the immunophenotyping experiments, since the homogeneity of the genetic background between pups is essential to compare their immune profiles, the MMc-depleted and control pups were genetically identical B6CF1 pups (F1 pups, Fig. 1B), unlike the backcrossed (B6CF1) B6 pups used for the depletion assays (F2 pups, Fig. 1A). As DT injections can lead to the production of antibodies (Buch et al., 2005; Rombouts et al., 2017), the control pups were born from DTR(−/−) dams such that DT injections could be performed without affecting the presence of maternal cells and to avoid biases in the immune profiles due to reactivity against DT. Unfortunately, as our detection method for maternal cells relies on the presence of the DTR gene, the numbers of MMc cells and their potential variability could not be estimated in these control pups.

### Detection of DTR(+/−) maternal cells in the spleen and thymus of pups

**Fig. 2.**
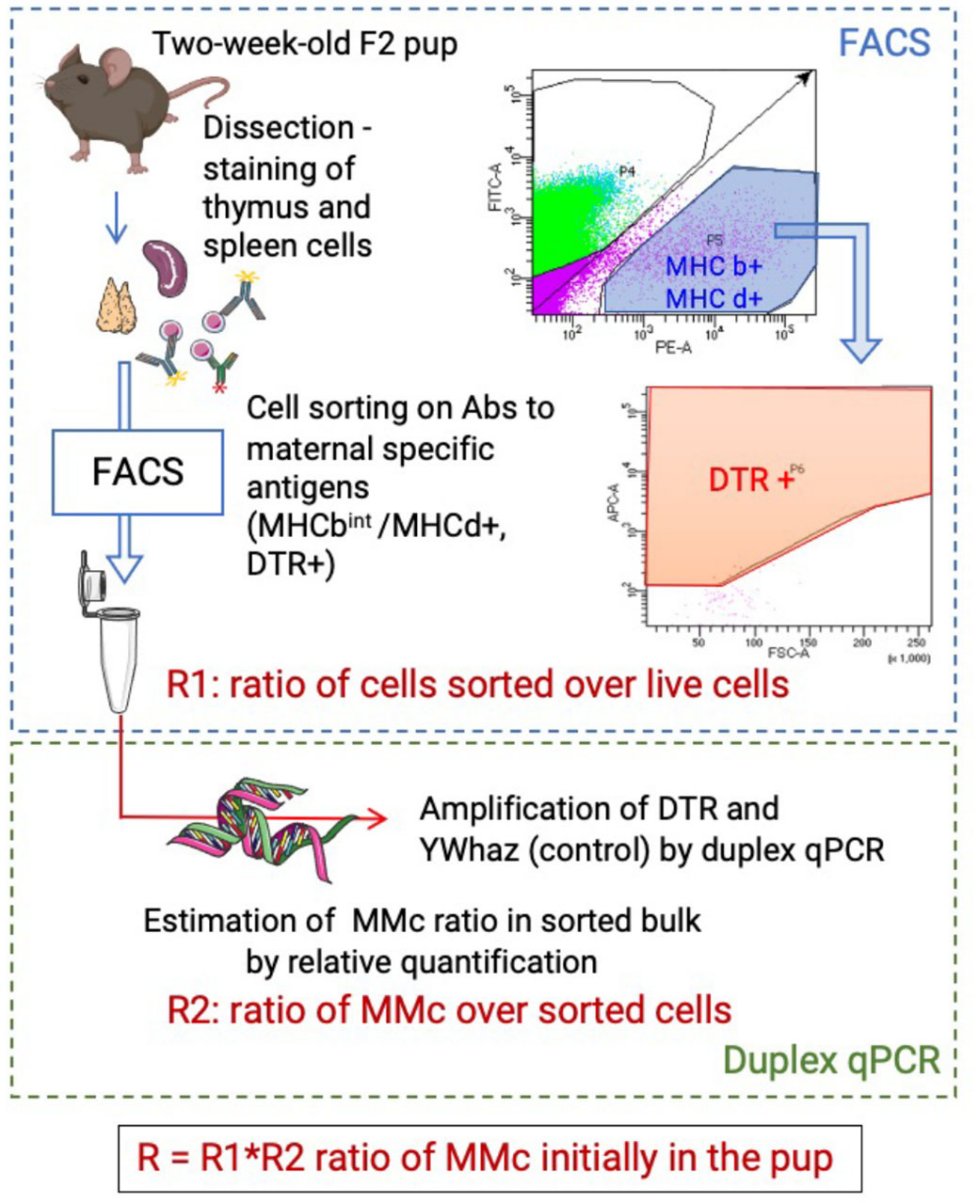
**Maternal cells**’ **detection pipeline.** Pipeline combining FACS sorting and TaqMan duplex qPCR for detection and estimation of maternal cells ratios R in the pups’ spleen and thymus. DTR: diphtheria toxin receptor. MHC: major histocompatibility complex. MMc: maternal microchimerism. Created with Servier Medical Art.

Considering the low frequency of MMc in the host, we attempted to improve the limiting sensitivity of the usual MMc detection methods by flow cytometry and qPCR (Eikmans et al., 2014; Thiele et al., 2014) by combining them (Fig. 2). Maternal cells were first enriched by flow cytometry cell sorting, and the ratio of cells sorted as MHCb^int^/MHCd+/DTR+ potential maternal cells over the live singlet cells R1 was recorded. The proportion of maternal cells among the sorted cells R2 was further assessed with qPCR by adapting the Pfaffl relative quantification method for DTR and a reference gene (Pfaffl, 2004). The initial proportion of maternal cells in the pups' organs R was calculated by factoring the ratios R1 and R2; normalized as the number of MMc per 10^7^ host cells. The sensitivity of this method was estimated to be under one cell in 3,000,000 cells using artificial mixes of DTR(+/−) MHC b/d cells among DTR(−/−) MHC b/b cells (Fig. S1).

**Fig. 3.**
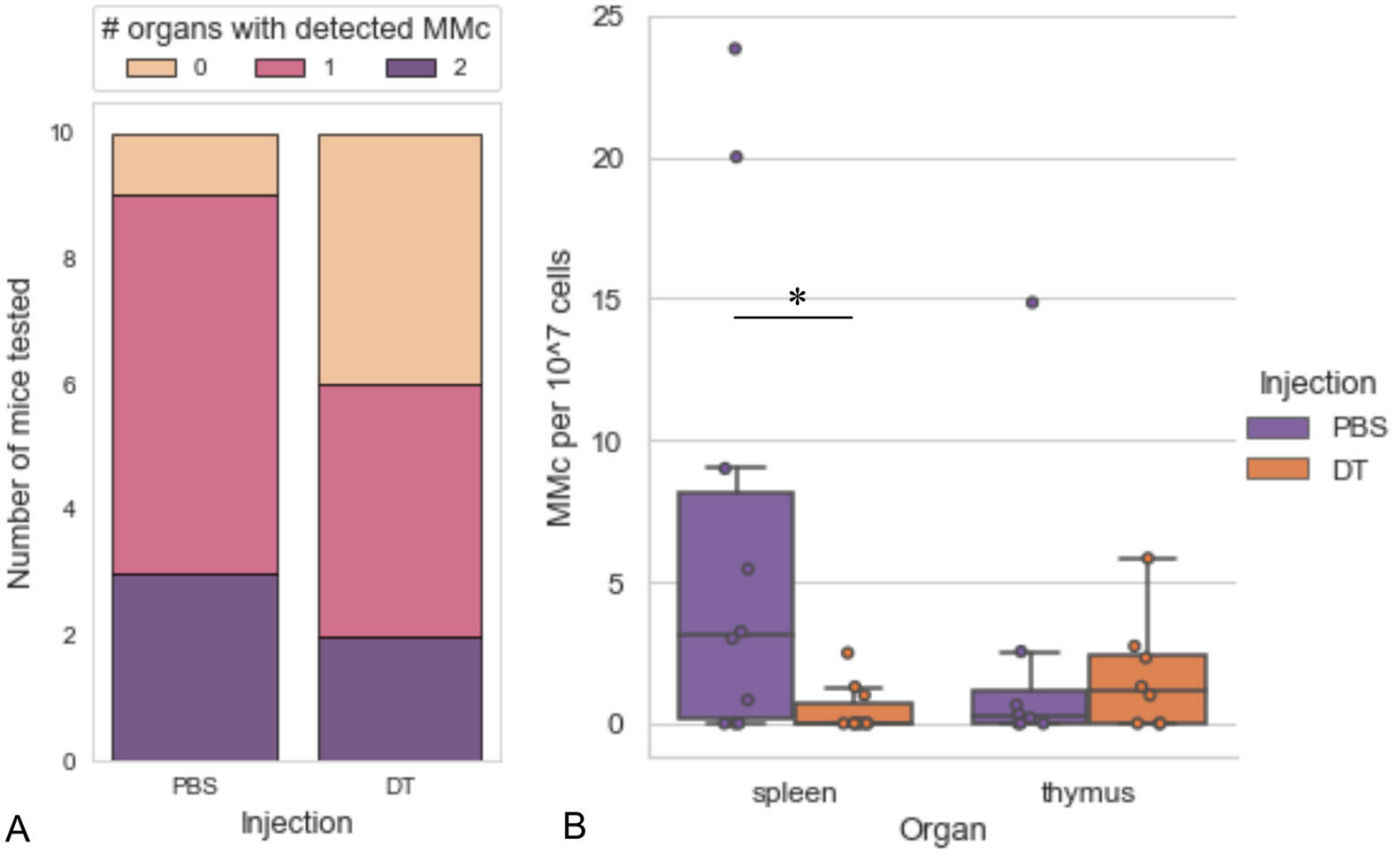
**Detection and depletion of maternal cells in the spleens and thymus of mouse pups.** (A) Numbers of F2 pups with the indicated number of organs positive for maternal cells out of the spleen and the thymus assayed by the FACS-qPCR pipeline following PBS or diphtheria toxin (DT) injections, *n*=10 mice for each group. (B) Estimated number of maternal cells per 10^7^ host cells in the spleens and thymus of F2 pups injected with DT (*n*=10 mice) or PBS (*n*=10 mice). The distribution of the data is represented with boxplots. Dots represent individual sample points. Brunner–Menzel's test: *P*=0.012.

This pipeline was applied to detect MMc in the pups’ largest lymphoid organs, the spleen and thymus. Maternal cells were detected in the spleen or thymus of as many as nine out of the 10 control pups (injected with PBS) assayed (Fig. 3A). Higher numbers of MMc cells were detected in the spleens compared to the thymus (Fig. 3B, PBS group). As previously reported (Dutta et al., 2017; Fujimoto et al., 2021), strong inter-pup variability in the numbers of maternal cells was observed.

### Depletion of maternal cells

To evaluate the depletion efficiency of DTR(+/−) maternal cells by DT injection in F2 pups (Fig. 1A), we compared the numbers of MMc cells in 2-week-old pups injected from postnatal day 3-5 with DT (*n*=10) or PBS (*n*=10, controls). Injections were carried out every other day until euthanasia to avoid further MMc originating from lactation (Ghosh et al., 2016). Although MMc cells were still detected in at least one organ in 60% of the DT-injected pups as can be seen in Fig. 3A, the mean MMc cell numbers in the spleens of pups injected by DT sharply dropped by a factor of 13.6 compared to the control pups. While the number of MMc cells was lower in the thymus than in the spleens in the controls, no significant difference in their number was observed in DT-injected pups (*P*>0.67) (Fig. 3B). Since the spleen, a major reservoir of immune cells, was substantially depleted of maternal cells, we decided to use this tissue for immune profiling in MMc-depleted conditions.

### Immunophenotyping MMc-depleted pups

**Fig. 4.**
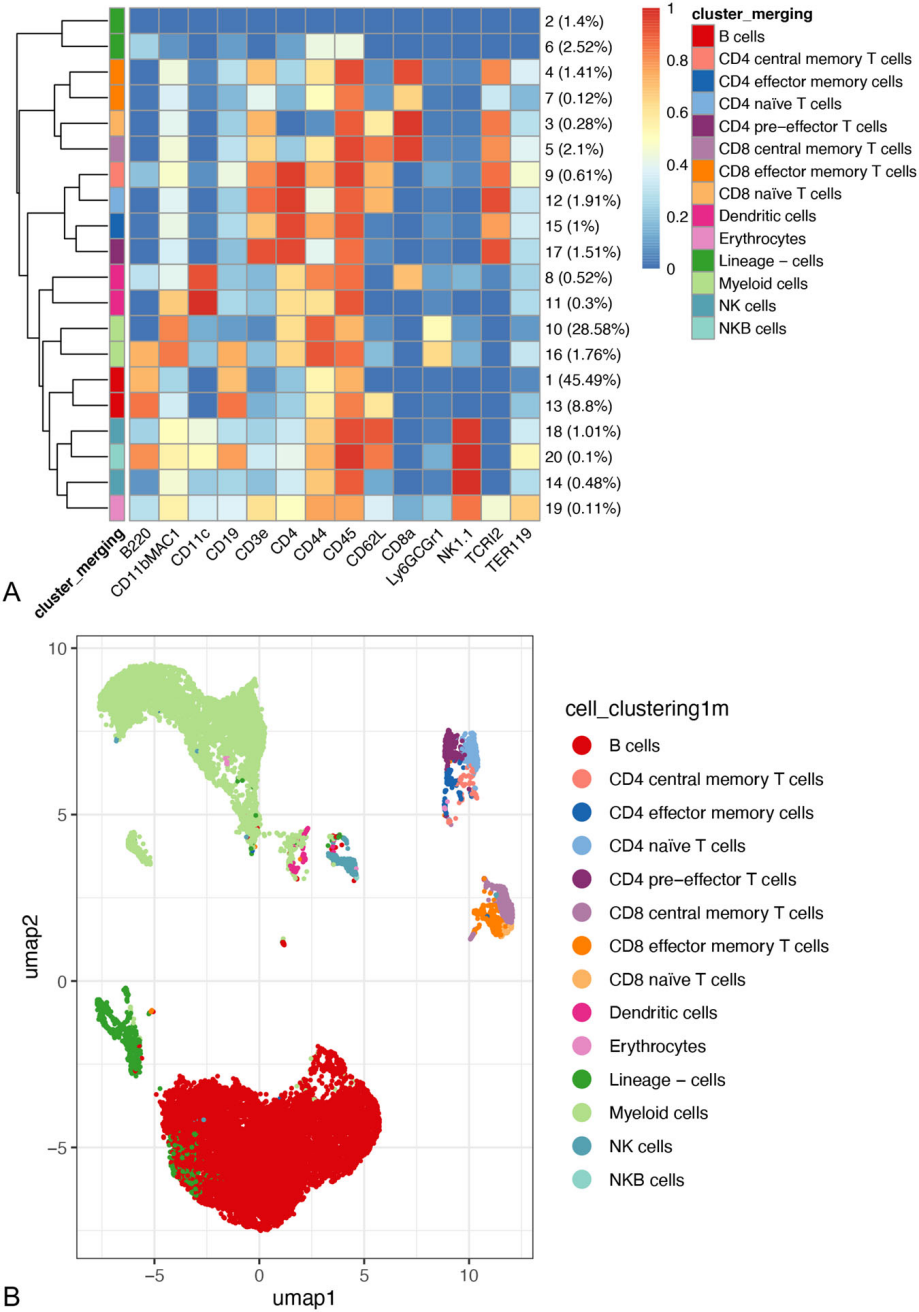
**Visual representation of the cell clustering performed by unsupervised meta clustering and manual merging.** (A) Heatmap representing the median of 0-1 transformed marker expression of the 16 markers across the initial 20 cell populations obtained with FlowSOM. The dendrogram on the left represents the hierarchical similarity between the 20 meta clusters (metric: Euclidean distance; linkage: average). The 14 merged clusters are identified by the color bar on the left of the heatmap. The 20 initial clusters are identified by their number at the right of the heatmap, with the relative proportion of each cluster over the total cell population indicated in brackets. The total cell population represents 141,174 cells per sample with *n*=10 mice. (B) UMAP plot based on the expression of the 16 immune cell markers. From each of the 10 mouse spleen samples, 2000 cells were randomly selected. Cells are colored according to the manual merging of the 20 cell populations obtained with FlowSOM into 14 populations.

**Fig. 5.**
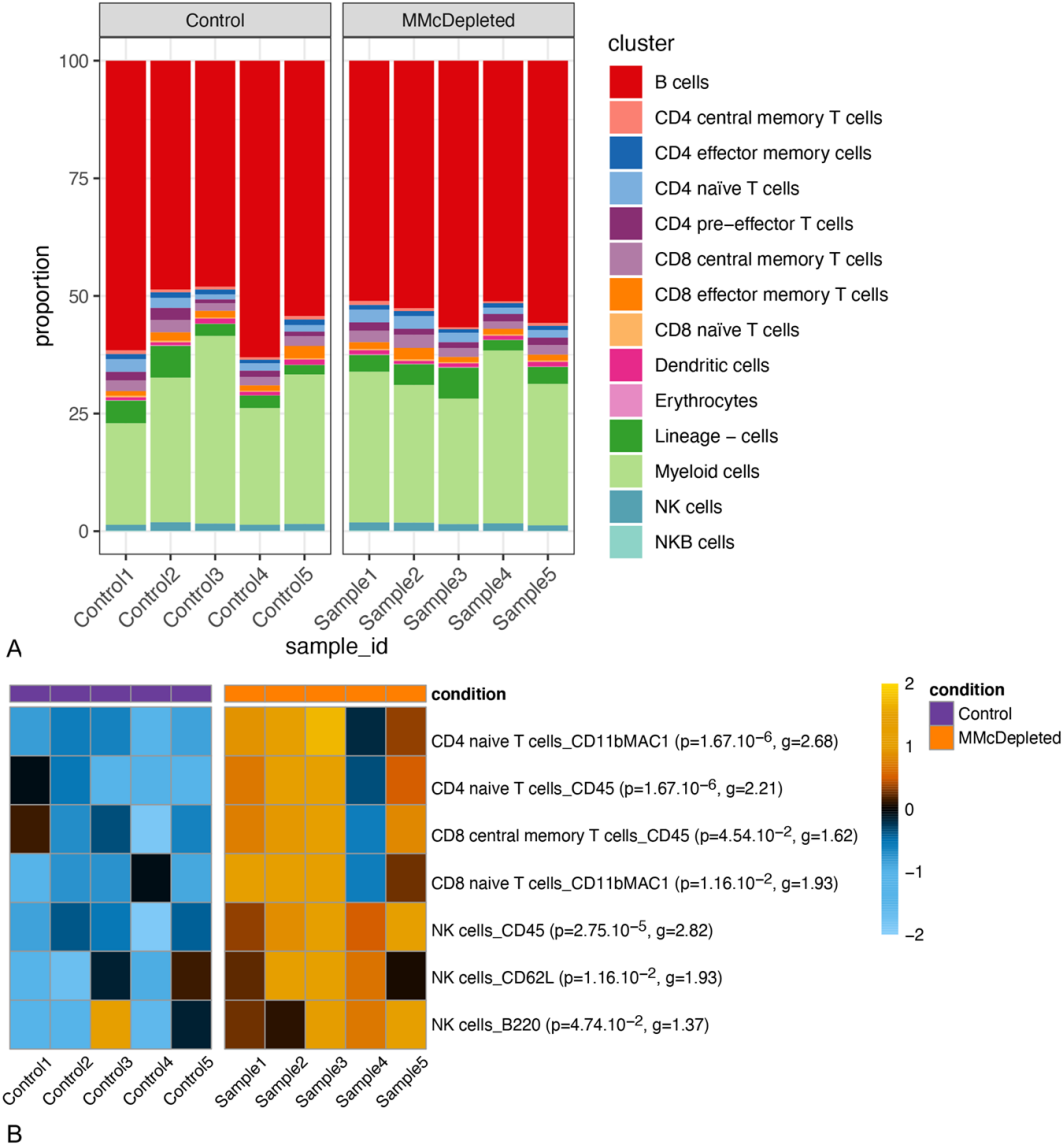
**Proportions of the immune cell populations and differential analysis of marker expression in these populations between splenic cells from reference pups (control, *n*=5) and pups depleted of maternal cells (MMc-depleted, *n*=5).** (A) Relative abundance of the 14 manually identified cell populations in each sample of the dataset, represented with a barplot, over the 141,174 subsampled cells per sample. (B) Heatmap of the normalized expression of the functional markers in the cell populations that are significantly differentially expressed between control and MMc-depleted conditions. Each median marker expression was normalized to have a mean of 0 and a standard deviation of 1. In parenthesis, value g indicates Hedges’ g measure of the effect size and the value *P* corresponds to the *P*-value for rejecting the general linear hypothesis of a null regression coefficient for the population marker expression corresponding to the condition.

To understand the influence of maternal cells on neonatal immune development, we compared the immune profiles of the spleens of MMc-depleted pups (MMc-depleted; *n*=5) and control pups (control; *n*=5) after DT injections with a mass cytometry immunophenotyping panel (F1 and control pups, Fig. 1B). Depletion of MMc was confirmed in the remaining spleen cells of the MMc-depleted samples (*P*=0.00068, Brunner–Menzel test). The mean number of MMc cells was 10.0-fold lower in these pups (1.08±0.80 MMc cell per 10^7^ cells; *n*=4) compared to PBS-injected genetically identical littermates (10.77±7.02 MMc cells per 10^7^ cells; *n*=3). Mass cytometry data were first clustered by unsupervised algorithms, and then cell clusters were manually merged and classified into cell populations according to expression profiles of lineage markers as presented in Fig. 4. For instance, the conventional T cells were identified based on CD3 and TCRβ expressions and clustered as helper CD4+ or cytotoxic CD8+ T cells. Within these groups, naïve T cells were defined as CD62L+CD44-, central memory T cells as CD62L+CD44+, effector/effector memory T cells as CD62L-CD44+ and pre-effector T cells as CD62L-CD44- (Nakajima et al., 2021; Sckisel et al., 2017). The heatmap in Fig. 4A shows the expression profiles used for the clusters’ merging and identification of cell populations. The dendrogram of Euclidean distances between the clusters (Fig. 4A, left side) and the visual representation of the clusters by UMAP (Fig. 4B) confirm the manual merging of the clusters to be satisfactory, as cells from related lineages clustered together. Differential analysis of the relative abundance of cell populations did not reveal any of the identified cell populations to be differentially represented among the MMc-depleted spleen cells compared to the control spleen cells (Fig. 5A). It is nevertheless noteworthy to point out the higher deviation within the control pups' immune profiles, both in terms of cell population proportions (Fig. 5A) and median marker expressions (Fig. S2, *P*=0.008 Wilcoxon rank-sum exact test). In the differential analysis of marker expression stratified by cell population (Fig. 5B), among the MMc-depleted samples, CD4+ and CD8+ naïve T cell populations had heightened expressions of CD11bMAC1, indicating recent activation (Christensen et al., 2001; Wagner et al., 2001). Additionally, the CD4+ naïve T cells and the CD8+ central memory T cells exhibited higher expression of the pan-hematopoietic cell marker CD45, which can be associated with activation (Petkov et al., 2020). All in all, the markers with an enhanced expression in some T cell populations in the MMc-depleted pups are linked with activated phenotypes.

The natural killer (NK) cells also showed greater expressions of markers CD62L, CD45 and B220 in the MMc-depleted samples (*P*<0.05, Fig. 5B). The markers CD62L and CD45 are markers of maturation in NK cells (Chiossone et al., 2009; Krzywinska et al., 2016), while the expression of B220 points toward relatively mature phenotypes (Guimont-Desrochers et al., 2012). These results suggest that maternal cell depletion leads to higher expressions of markers linked with activation and maturation in some T cell and NK cell populations.

## DISCUSSION

Maternal cells transferred during pregnancy engraft in the fetus where they are reported to promote fetal tolerance toward themselves and fetal immune development (Stelzer et al., 2021). However, whether their persistence after birth is coincidental or can be explained by further functions of MMc in postnatal life is still under debate; with the enhancement of immune responses (Balle et al., 2022) and cross-generational reproductive fitness (Kinder et al., 2015) postulated as some of these functions. In this study, we hypothesize a new postnatal role of maternal cells in suppressing the activation of neonatal NK and T lymphocytes.

Using the iDTR strain (Buch et al., 2005), we developed a novel model for postnatal maternal cell depletion by diphtheria-toxin (DT) injection in wild-type pups born from diphtheria-toxin receptor heterozygous dams. The depletion of maternal cells in the spleens was confirmed with our FACS-qPCR maternal cell detection method. We did not observe a DT-induced MMc depletion in the thymi−albeit containing less MMc than the spleens−in line with the reported difficulty of thymic cell ablation by DT injection with this iDTR system (Buch et al., 2005), which was explained by either low DTR expression by thymic cells or poor delivery of DT to the thymus.

Since our and previous studies (Dutta et al., 2017; Fujimoto et al., 2021) reported a high inter-pup variability in the number of maternal cells in pups, it is possible for the number of maternal cells in the control pups of the immunophenotyping experiment to largely differ. Whether this putative variability in MMc cell numbers in the controls compared to the MMc-depleted samples could be a factor in the observed higher deviation observed within the control pups’ immune profiles remains to be verified. Moreover, if some of these control pups naturally host a low number of maternal cells, this might offset the observed significance of some MMc depletion-induced immune profile differences in our analysis.

Immunophenotyping the MMc-depleted spleens of two-week-old pups revealed higher expressions of markers linked to activation and maturation by NK cells and T cells. Both naïve CD4+ and CD8+ T cells exhibited higher expressions of CD11b – the marker for recently activated T cells (Christensen et al., 2001; Wagner et al., 2001). The marker CD45, which was linked with activated expression profiles (Petkov et al., 2020) and is upregulated on mature memory CD8+ T cells (Cho et al., 2016), was more highly expressed in the MMc-depleted samples in the CD4+ naïve T cells and the CD8+ central memory T cells. Therefore, MMc cells might play a part in suppressing the early activation and further maturation of neonatal T cells. Among the natural killer cells, the increased expression of B220 in MMc-depleted samples points toward either prior activation of the NK cells (Juelke et al., 2010) or an enrichment in the highly proliferative pre-mNK (pre-mature natural killer) intermediate in NK-cell differentiation (Guimont-Desrochers et al., 2012). Together with the higher expression of CD45 and CD62L, markers of maturation in NK cells, this expression profile indicates an enhanced intermediate stage of maturation for NK cells in MMc-depleted pups. This suggests maternal cells may also contribute to suppressing NK cells maturation.

At first glance, these results appear to be in disagreement with previous studies suggesting MMc enhances immune development, in particular T cell immunity (Balle et al., 2022; Kinder et al., 2017; Stelzer et al., 2021). Notably, MMc was reported to promote fetal CD8+ T cell expansion and immunity against viral infections (Stelzer et al., 2021), such that we expected to observe fewer and more immature T cells in our MMc-depleted samples. Surprisingly, not only did we not observe such phenomenon, instead, we noted an increased in marker expression related to activation in the naïve T cells. This apparent discrepancy may stem from different timings in the depletion of maternal cells between the studies. As such, maternal cells could be playing a prominent role in the maturation and expansion of the nascent immune system *in utero* (El Haddad et al., 2021), which would be balanced postnatally with a function in regulating cell-mediated immunity responses.

After birth, the neonatal immune system swiftly shifts to mature hematopoiesis and can mount fully developed reactions in some circumstances, while immune responses are generally reported as blunted (Adkins et al., 2004), raising questions about how this flexible responsiveness is regulated. We hypothesize a potential role of maternal cells in regulating the activation of T and NK cells in the pup, possibly to avoid broad reactivity against foreign and self-antigens and instead promote tolerance. Indeed, increased expression of NK cell activation markers could both predispose to and respond to allergen sensitization (Altman et al., 2018), and regulate the concomitant T cell activation upon allergen exposure (Gerberick et al., 1997; Wingett and Nielson, 2003). This proposition of maternal cells suppressing deleterious neonatal immune reactivity is corroborated by the observation of lower rates of asthma, partially caused by NK and T cell mediated hypersensitivity (Karimi and Forsythe, 2013; Rottem and Shoenfeld, 2003), in human infants carrying maternal cells (Thompson et al., 2013). On the other hand, disruption of this presumed tolerogenic function of MMc cells might lead to harmful consequences, such as alloimmune conflicts (Bastian et al., 1984; Müller et al., 2001) and loss of self-tolerance leading to autoimmune disorders (Irie et al., 2009; Muraji et al., 2009).

Despite these promising results, one should bear in mind the restriction of the immunophenotyping to cells from a single lymphoid organ − the spleen − at a single developmental timepoint. The extent of maternal cell depletion in our DT model needs to be assessed more comprehensively, and more lymphoid organs could be included. Being able to estimate the number MMc cells in the controls would be valuable considering their potential variability may hinder the observation of the immune effects of MMc depletion and to evaluate if differences in the amounts correlate with immune profiles. Further experiments investigating immune reactivity to foreign antigens of pups depleted of maternal cells are needed to confirm the proposed functional role of MMc in immune regulation.

In summary, when depleting maternal cells in pups after birth, we observed increased expression of markers of activation and maturation in the splenic NK and T cells. Expanding presumed maternal cells’ role in fetal immune system maturation, we suggest that the postnatal functions of maternal cells may include the suppression of NK and T cells, responses. Questions remain whether this phenomenon can be translated to humans, considering the species’ distinct immune maturation timings, and if it could regulate infantile hypersensitivity reactions such as allergic asthma.

## MATERIALS AND METHODS

### Mice

Mouse care and all experimental procedures were performed according to The University of Tokyo institutional guidelines and experiments approval was obtained from the Institutional Animal Care and Use Committee of The University of Tokyo. B6 (C57BL/6JJcl), Balb/c (BALB/cByJJcl), CAG-Cre [C57BL/6-Tg(CAG-cre)13Miya (Sakai and Miyazaki, 1997), strain number RBRC09807] and iDTR [C57BL/6-*Gt(ROSA)26Sor^tm1(HBEGF)Awai^*/J (Buch et al., 2005), strain number 007900] *Mus musculus* were acquired from Nihon Clear Japan, RIKEN and The Jackson Laboratory, respectively. Diphtheria Toxin Receptor (DTR) heterozygous mice on the B6 background were obtained by mating iDTR males and CAG-Cre females and genotyping the pups for the absence of CAG-Cre (based on RBRC09807 genotyping protocol). Hybrid B6CF1 DTR(+/-) females were subsequently obtained by crossing B6 DTR females with Balb/c males and genotyping for the presence of DTR (based on JAX 007900 protocol).

### Depletion of maternal cells in pups

To obtain semi-allogeneic wild-type pups from DTR dams, (B6CF1)B6 offspring of B6CF1 DTR(+/−) females and B6 males (F2 pups, Fig. 1A) and B6CF1 offspring of B6 DTR females and Balb/c males (F1 pups, Fig. 1B) were genotyped from toe clippings at postnatal day 2-3 and selected against the presence of DTR gene for F1 and F2 pups and against H2-Dd allele for F2 pups (primers by Dutta et al., 2017). To eliminate DTR maternal cells, the pups were injected intraperitoneally with DT or PBS (control) every other day from postnatal days 3 to 5; pups aged 3-6 days were anesthetized by hypothermia over ice and pups aged 7-15 days were anesthetized using isoflurane gas (Fujifilm). Diphtheria toxin (Sigma-Aldrich) at a concentration of 5 ng/μl was injected to the amount of 8 ng/g of body weight (averaged among the injected littermates) as in (Bar-On and Jung, 2010), and the matching volume of PBS was injected in the control littermates. Pups were euthanized by cervical dislocation following isoflurane anesthesia at postnatal day 13-15 and the spleen and thymus were harvested for downstream analysis. Three male and seven female pups were used in each of the sample (DT-injected pups) and control (PBS-injected pups) groups. A two-way ANOVA analysis over the maternal cells frequencies data from all pups and organs assayed, modelling maternal cells frequencies as a response to the variables: treatment, sex, organ and treatment*sex (interaction term), showed no interaction between the treatment and sex variables [f(1)=1.07, *P*=0.308] and confirmed the statistically significant difference in maternal cells frequencies led by treatment type [f(1)=4.87, *P*=0.035], but not by the factor sex [f(1)=2.95, *P*=0.095].

### Cell suspensions, staining and flow cytometric analyses

To assay maternal cells’ presence in the pups’ lymphoid organs, cell suspensions were prepared from postnatal day 13-15 pups’ spleen and thymus by mechanical dissociation using 70 μm nylon mesh (Axel). Cell suspensions were treated with red blood cells lysis buffer (ammonium-chloride-potassium) and washed in staining buffer [HBSS(-), 2 mM EDTA, 1% BSA]. Single cells were stained in a 96-well plate at a concentration of 3-5×10^6^ cells per well. Cells were stained simultaneously with LIVE/DEAD Fixable Near-IR Dead Cell Stain Kit (eBioscience) at 1 μl/well, H2Kb-FITC (BioLegend clone AF6-88.5) and H2Dd-PE (BioLegend clone 34-2-12) at a concentration of 1/75th for 30 min at 4°C; fixed and permeabilized using Cytofix/Cytoperm kit (BD Biosciences); blocked with Fc Receptor Binding Inhibitor Polyclonal Antibody (eBioscience) at 20 μl/well for 15 min and stained intracellularly with HB-EGF-APC (R&D Systems clone 125923) at a concentration of 1/10th for 30 min at room temperature. The following day, 20-30×10^6^ cells per sample were analyzed and sorted on a fluorescence-activated cell sorter (BD FACSAria III) using the BD FACSDiva software (Becton-Dickinson). Sorting was performed by gating for live singlets and maternal cells’ markers on gates H2Kb^+^H2Dd^-^Hbegf^+^ and H2Kb^int^H2Dd^+^Hbegf^+^ for F1 and F2 pups, respectively, and the ratio of sorted cells over live cells R1 was recorded.

### DNA lysis and quantitative PCR analyses

Sorted cells were lysed and the ratio R2 of maternal cells in this enriched sorted bulk was assessed with TaqMan duplex qPCR on the maternal-specific gene DTR and the endogenous control gene YWhaz by quantification to a positive control (DTR+ cells) in a protocol partially based on (Drabbels et al., 2011). Quantitative PCR analyses were performed using the Brilliant III ultra-fast qPCR master mix (Agilent) for TaqMan duplex qPCR amplification of the DTR gene and a reference gene *YWhaz*. Reactions were performed in 20-40 μl with 2-4 μl cell lysis solution and sense primers/antisense primers/probes at concentrations of 100/150/100 nM for DTR and 300/150/200 nM for YWhaz. PrimerBlast was used to design the forward and reverse PCR primers (purchased from Thermo Fisher Scientific) and Primer 3 Plus and PrimerQuest (IDT DNA) were used to design the probes (purchased from IDT DNA): DTR sense primer, 5′-CCTCCCAGTGGAAAATCGCT-3′; DTR antisense primer, 5′-ACATGAGAAGCCCCACGATG-3′; probe DTR, 5′-FAM-TGGTGCTGT-ZEN-CATCTGTCTGTCTGC-3′-BkFQ; YWhaz sense primer, 5′-AATTGTCTCCTTATTCCCTCTTGGC-3′; YWhaz antisense primer, 5′-GTCCACTTCAATTGTTGAGGAGTAA-3′; probe YWhaz, 5'-HEX-CATCCTCAG-ZEN-CCACCTCCCACATTT-3′-BkFQ. The primers and probes concentrations were determined by running concentration matrices and singleplex/duplex metrics. The qPCR program used an initial temperature of 95°C for 3 min, followed by 45 amplification cycles ran for 15 s at 95°C and 20 s at 60°C. The amplifications were performed on a Stratagene Mx3000P qPCR system (Agilent) and data were collected with Mx Pro qPCR software (Agilent). For calibrators and amplification efficiencies determination, a five-point two-fold standard curve of simultaneously sorted DTR+ cells was run alongside the samples, and a negative control sample was included to control against false positives. The ratio of DTR cell equivalent in the DNA of each well was calculated using the Pfaffl relative quantification method for DTR, relative to the *YWhaz* reference gene amplification and a DTR+ positive control calibrator. The ratio R2 of DTR cells (corresponding to maternal cells) present within the bulk sorted sample was calculated across all wells corresponding to that sample.

### Immunophenotyping

To compare immune profiles in pups with or without maternal cells, B6CF1 control pups from a B6 female and a Balb/c male (*n*=6; control), and F1 pups (Fig. 1B) from a B6 DTR(+/−) female and a Balb/c male (*n*=5; MMc-depleted) were injected intraperitoneally every other day from postnatal day 3 with DT as described above. The pups from each group were obtained from two batches, from the first litter of age-matched females for each batch (8 weeks old for the first batch, 16 weeks old for the second batch), and were all female. One control pup from the first batch was excluded from the analysis due to having considerate average Euclidian distance with the other control samples based on median marker expression D=1.99 compared to the other control samples with each other when excluded (0.99±0.15 s.d., *n*=5). The dissimilarity of this sample can be observed as Control 2 in the diagnostic MDS plot and in the boxplot of the average distances within the conditions (control or MMc-depleted) in Fig. S2, prior to its exclusion and the control mice re-numbering. Cell suspensions were prepared as described above from the spleens of the 15-day-old pups, were frozen at −80°C in Cell Banker 1 Plus, thawed and stained on the same day across experimental groups for each batch. The cells were stained using Maxpar Mouse Sp/LN Phenotyping Panel Kit (Fluidigm) containing the following Abs: Ly6G/C (RB6-8C5), CD11c (N418), CD69 (H1.2F3), CD45 (30-F11), CD11bMAC1 (M1/70), CD19 (6D5), CD25 (3C7), CD3e (145-2C11), Ter-119 (TER119), CD62L (MEL-14), CD8a (53-6.7), TCRβ (H57-597), NK1.1 (PK136), CD44 (IM7), CD4 (RM4-5), B220 (RA3-6B2); with Cisplatin 198Pt used as a dead cells’ marker. Cells were fixed in 1.6% PFA, transported at 4°C to Fluidigm Tokyo and analyzed on the Helios mass cytometry system. Mass cytometry data were first preprocessed with R using the package openCyto (Finak et al., 2014) to select live single cells by gating on DNA1+/DNA2+ and on 198Pt- with a quantile gate based on manually measured viability data pre-fixation. The samples were subsampled to 141,174 preprocessed events to contain a homogeneous number of events. The two batches of data were integrated and normalized using cyCombine (Pedersen et al., 2022). Further analyses were adapted from the Bioconductor-based CyTOF Workflow pipeline (Nowicka et al., 2019). The 16 markers’ expression data were transformed with arcsinh (hyperbolic inverse sine) with cofactor 5. After unsupervised over-clustering using the FlowSOM (Van Gassen et al., 2015) and ConsensusClusterPlus (Wilkerson and Hayes, 2010) packages, clusters were further manually merged and identified into cell populations based on median marker expression data. The merged clusters were visually inspected by Uniform Manifold Approximation and Projection (UMAP) plot and heatmaps (Fig. 4). The differential analysis of clusters’ abundance and clusters’ markers’ expression between the two conditions (control and MMc-depleted) were performed using generalized linear mixed models (GLMM), where the random effect was defined by the sample ID. As batch effects were still substantial in these results, the differentially expressed marker-cluster combinations selected as significant were filtered on having a higher Hedge's g absolute value considering the condition (MMc-depleted versus control) compared to the batch (Batch1 versus Batch2).

### Statistical analysis

For comparing the differences in the means of detected maternal cells, Brunnel–Menzel's non-parametric test for unequal variances was applied (Brunner and Munzel, 2000). Measures of MMc cells numbers in the text are expressed as means±s.e.m. For the differential analysis of immunophenotyping data, generalized linear mixed models with random effects were used and the null hypothesis tested the regression coefficient for the condition (control or MMc-depleted) to be equal to 0, the *P*-value indicates the probability of observing a similar difference between the two conditions if the null hypothesis was true. A *P*-value <0.05 was considered significant unless otherwise specified. The Hedges’ g, noted g, statistic was used to measure the effect size for small sample sizes.

## Supplementary Material

10.1242/biolopen.059334_sup1 Supplementary information
