# Supplementary material for: Postnatal depletion of maternal cells biases T lymphocytes and natural killer cells’ profiles toward early activation in the spleen
Source: Biol Open. 2022 Nov 9;11(11):bio059334. doi: 10.1242/bio.059334 (PMC9672855; doi:10.1242/bio.059334)
Supplement: Supplementary information [file biolopen-11-059334-s1.pdf]

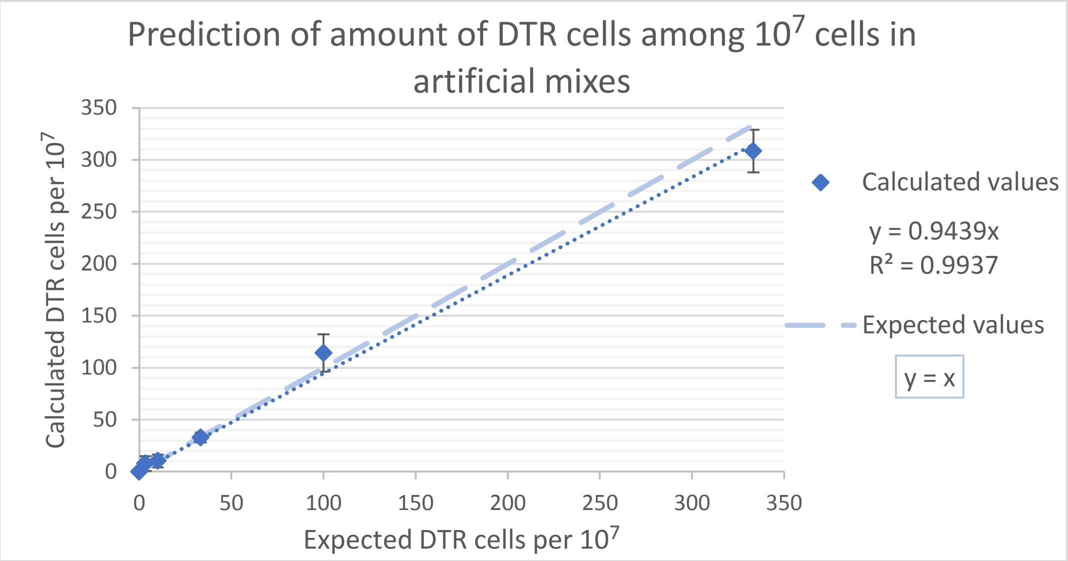

Fig. S1. Estimation of the ratio of DTR(+/-) MHC b/d cells in WT cells by the FACS-qPCR pipeline in artificial mixes. Averaged calculated amount of DTR(+/-) MHC b/d cells per 10^7 DTR(-/-) MHC b/b cells in 5 artificial mixes ranging from 1 DTR(+/-) MHC b/d cell in 30,000 to 3,000,000 DTR(-/-) MHC b/b cells, for a total of 15.10^6 to 20.10^6 cells per mix. Results were replicated 3 times. Error bars indicate standard deviations. DTR: diphtheria toxin receptor.

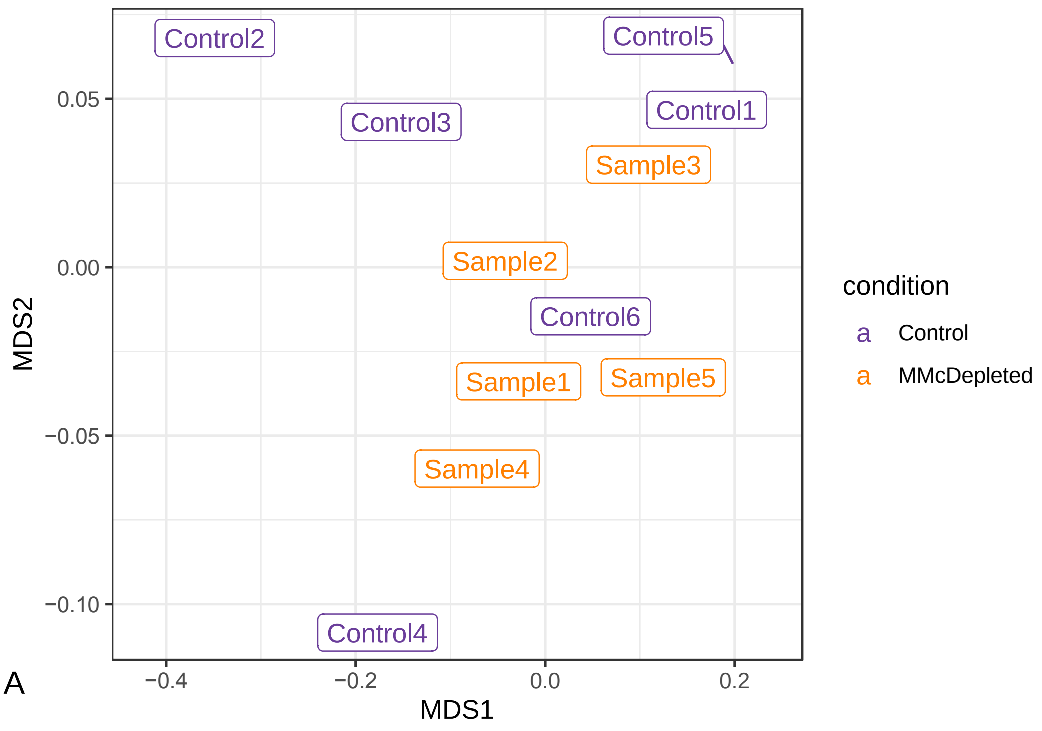

A

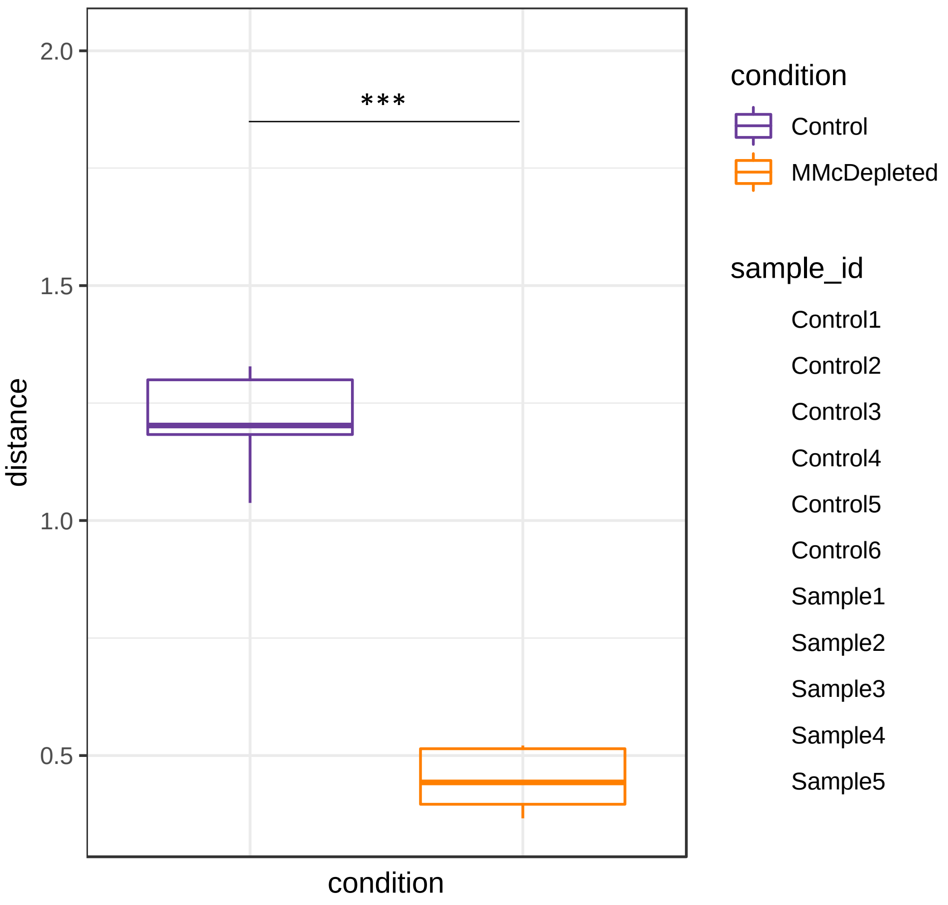

B

Biology Open • Supplementary information

Fig. S2. Distances between samples based on marker expression

(**A**) Multiple Dimension Scaling (MDS) plot for the control and MMcDepleted samples based on median markers expression of the 16 markers across 141,174 subsampled cells for each sample. (**B**) Boxplot of the average Euclidian distance of each sample with the other samples in the same condition group (Control or MMcDepleted), based on median markers expression of the 16 markers across 141,174 subsampled cells for each sample. Wilcoxon rank sum exact test: W = 30, p-value = 0.004329. n=6 for the control group and n=5 for the MMcDepleted group. This data is prior to the exclusion of the Control 2 sample as an outlier and the control pups renumbering. After exclusion of the Control 2 sample, Wilcoxon rank sum exact test: W = 25, p-value = 0.007937.
